# The contribution of Australian fathers in getting food on the table among families with young children

**DOI:** 10.1017/S1368980023001817

**Published:** 2023-12

**Authors:** Konsita Kuswara, Helen Vidgen, Penelope Love, Rachel Laws, Karen J Campbell

**Affiliations:** 1 School of Exercise and Nutrition Sciences, Institute for Physical Activity and Nutrition (IPAN), Deakin University, Burwood, VIC Australia; 2 Queensland University of Technology (QUT), School of Exercise and Nutrition Sciences, Faculty of Health, Victoria Park Road, Kelvin Grove, QLD, Australia

**Keywords:** Fathers role, Cooking, Food literacy, Young children, Family meals, Food agency

## Abstract

**Objective:**

To explore how fathers with young children contributed to healthy home food provisioning and the factors enabling or inhibiting their involvement in family food tasks.

**Design:**

Cross-sectional study using purpose-designed online survey. The survey assessed the level of responsibilities and practices in family food tasks, food agency (Cooking and Food Provisioning Action Scale), and use of resources to support involvement in family food tasks. Data collection took place over 3 weeks in November–December 2020 when various COVID-19-related restrictions were in place. Descriptive and regression analyses were used to assess psychosocial factors influencing responsibilities in family food tasks and food agency.

**Setting:**

Online survey.

**Participants:**

Included in the analysis were 435 Australian fathers with children aged under 5 years.

**Results:**

Between 75 and 77 % of fathers in this study reported having at least half of the responsibilities in meal planning, shopping, and cooking. Health was frequently considered when deciding what to eat, but few used nutrition or food labels when shopping, tried new recipes or modified recipes to make them healthier. Involvement in family food tasks was promoted by a higher food agency, but time spent in employment was a significant barrier to reported food agency and greater involvement in food tasks. There was a high interest in resources to support healthy home food provisioning.

**Conclusions:**

The findings suggest the need to consider father-specific strategies to overcome time barriers and opportunities to enhance their capabilities for healthy home food provisioning.

The early childhood period (0–5 years) is an opportune time to cultivate children’s healthy eating habits. Parents have an important influence in promoting these habits by shaping the family food environment^(1)^. Studies examining parental influence on children’s eating habits in early childhood have predominantly focused on mothers^(2)^. While mothers often have a greater responsibility in family food-related tasks and child feeding^(3)^, it is likely that fathers could influence family food practices and diet through the way they engage (or not) with healthy food provisioning at home^(4)^. Child development, including nutrition, is shaped by all layers of the Social Ecological Model of Health^(5)^. Fathers are a distinct and essential part of the microsystem influencing the young child’s development through their interactions with the child and the child’s mother, for example, in responsive caregiving, role modelling, and providing a nurturing environment^(6)^. Taking a father-inclusive approach could enhance the effectiveness of family-based programmes to improve child health outcomes^(7)^.

There is growing evidence of fathers’ influence on young children’s eating behaviours, over and above the influence of mothers^(8,9)^. A systematic review of predominantly observational studies reported positive correlations between fathers’ weight, dietary intake and feeding practices, and their children’s food intake, weight and eating behaviours^(8)^. For example, children were more likely to be affected by overweight or obesity if the father experienced overweight or obesity; fathers’ use of control and restriction of food was associated with higher child weight, but reinforcement of healthy eating promoted children eating more nutrient-dense foods^(8)^. The association between father and young children’s eating behaviour and dietary intake is likely mediated partly through role modelling and feeding practices^(9)^. Longitudinal evidence from a cluster-randomised controlled trial showed that fathers’ influence on their child’s dietary intake commenced in the first 2 years of life and continued through to 5 years of age, even after adjusting for maternal factors^(10)^. Given that the majority of children aged under 5 years live in two-parent households^(11)^, and the likely influence of both parents, it is important to understand how to engage and support both parents to promote healthier eating habits in young children.

Engaging fathers in home food provisioning is likely to be important with changing societal trends encouraging more women in paid work. Recent Australian labour force data (2022) showed that the majority (71 %) of couple families with dependent children have both parents in employment, and increasingly, both parents in full-time employment (31 %)^(12)^. Concurrently, families where the father was employed and the mother was not have steadily declined from 37 % in 1990 to 20 % in 2022^(11)^. While overall women’s participation in employment has increased, the pattern remains gendered in couple households with children aged under 5 years, where more women work part-time (39 %) compared to men (13 %)^(12)^. In contrast, fathers’ employment patterns and work hours changed little by the age of the youngest child^(12)^.

The gendered pattern among Australian families with young children is further reflected in the time spent on home duties. Analysis of nationally representative longitudinal data from the 2018 Household, Income and Labour Dynamics in Australia (HILDA) Survey showed that in coupled households with dependent children, women spent more than double the time than men on housework and childcare (54 *v*. 26 h/week) and less than half the time on paid employment (22 *v*. 47 h/week)^(13)^. The gap in unpaid housework between men and women widens significantly when children are born and persists as they grow older^(13)^. International studies found similar trends in other Western countries except for Nordic countries^(14,15)^. Becoming a parent seems to promote a long-term pattern of a highly gendered division of unpaid labour driven by changes in identity upon becoming parents, and social structures that favour traditional gendered roles^(16)^. Social and economic challenges, including workplace norms, the gender pay gap and career disruption, may discourage fathers from taking parental leave and may further entrench traditional gender roles upon childbirth^(17)^. These studies suggest that fathers of young children are less likely to be involved in family food tasks at a time when their engagement could have the most influence on their young children’s eating habits.

Fathers’ documented lack of engagement in family food tasks, including planning, procuring and preparing meals, has been associated with perceptions of food tasks as ‘women’s work’^(18)^ and a lack of capacities^(19)^. Qualitative studies suggest that Australian fathers perceived they have an important role in sharing the responsibilities of providing for and role modelling healthy lifestyle habits for their young children^(19,20)^. However, perceived lack of work flexibility, low levels of cooking skills and enjoyment were barriers to greater involvement in family food tasks^(19)^. Furthermore, fathers felt they lacked the knowledge to respond to their children’s nutrition and physical activity needs and were unsure how to access credible nutrition information^(20)^. These studies suggest that rather than being disengaged, fathers may be motivated and committed but need support in managing barriers around time, knowledge, skills and confidence to provide a healthy food environment in the home.

Enabling a healthy home food environment includes possessing the ability to plan meals, manage resources for food, select healthy foods, prepare and eat healthy meals, herein referred to as ‘family food tasks’^(21)^. Executing family meals require a series of cognitive and physical tasks that are interrelated and dynamically influenced by the people involved, their beliefs and feelings and the outcomes of the food work^(22)^. An individual’s food agency, that is internal motivation, knowledge and skills to complete the necessary steps to produce the meals envisioned within a given context, is an important factor in the performance of family food tasks^(23)^. Increased competencies to perform family food tasks have been linked with greater abilities and confidence in healthy home food provisioning^(24)^.

Previous studies examining fathers’ behaviour around family food tasks have focused on their participation in generic housework (including food shopping and cooking)^(3)^ and their roles and influence on family food practices^(4)^. However, no studies have examined their practices to support *healthy* food provisioning. This study aims to explore the family food tasks performed by fathers and the barriers and enablers to fathers contributing to healthy family food provisioning.

## Methods

### Study design and participants

This study used a cross-sectional survey administered online using Qualtrics (Provo, UT). The survey measured perceptions and views of responsibilities in family food tasks, practices in family food tasks, food agency, physical barriers to performing food tasks, interest in resources to support family food provision and sociodemographic details. Recruitment was conducted through paid Facebook^TM^ advertising from 10 November to 4 December 2020. During this time, various COVID-19-related restrictions were in place. The advertisement targeted males aged 18 years and older in Australia. Interested participants clicked on the study link in the advertisement and were directed to determine their eligibility: 18 years and older, expecting a child or whose oldest child was aged under 6 years. Eligible participants were then presented with detailed information about the study and provided consent to participate online. The survey took approximately 10 min to complete. At the end of the survey, participants could choose to go in a draw to win a $100 gift voucher from Bunnings^TM^ hardware store.

Using the principle of at least ten events per variable for logistic regression analysis^(25)^, and accounting for nine independent variables of 2–3 categories each, the minimal sample required for this study was 270.

### Measures

#### Perceptions and views of responsibilities in family food tasks

To assess perceived responsibilities in family food tasks, we purpose-designed six questions asking participants if they had any responsibilities in planning/shopping/preparing family meals. Response options included ‘Yes, all/a lot/equal/a little responsibility’ or ‘No responsibility’. Participants were also asked to indicate what they thought about their responsibilities for each task with response options including ‘I would like to be more involved’, ‘My current responsibilities are enough’ and ‘I have too many responsibilities and want to share’.

This study was conducted during the COVID-19 pandemic when various degrees of lockdowns were in place. To determine usual practice, participants were asked if their responsibilities around family food tasks had changed because of COVID-19-related restrictions with response options of ‘increased/fewer responsibilities’ or ‘no change’.

#### Practices in family food tasks

Practices in family food tasks were measured using an eleven-item tool previously developed to evaluate a food literacy programme in Western Australia (Food Sensations for Adults)^(26)^. Participants were asked how often they had planned meals, managed resources for food, selected or prepared healthy meals in the home (‘never, sometimes, most of the time, always’). Responses were scored 1 (‘never) to 4 (‘always’), multiplied by the factor loading for each question and summed to calculate the factor score for each of the three sub-scales, ‘Plan and Manage’, ‘Selection’ and ‘Preparation’^(26)^.

#### Food agency

Food agency was measured using the previously validated Cooking and Food Provisioning Action Scale (CAFPAS) tool^(27)^ consisting of twenty-eight items measuring self-efficacy (thirteen items, e.g. ‘I feel limited by my lack of cooking knowledge’), attitude (ten items, e.g. ‘I find cooking a very fulfilling activity’) and structural/social barriers (five items, e.g. ‘My job responsibilities prevent me from having the time to prepare meals’). Participants were asked to indicate how much they agreed with the statements on a seven-point Likert scale. The tool was modified from a seven-point to a five-point Likert scale (strongly disagree (1)–strongly agree (5)) to be consistent with other questions in this study.

As per CAFPAS protocol, the score of each subscale was determined by reversing negatively worded items, adding the coded response items (1–5) and dividing by the standard deviation. The overall food agency score was the sum of standardised scores of all three subscales. Higher scores indicate a greater *capacity* to complete the necessary steps to produce the meals desired. The (unstandardised) mean score per item was also calculated by adding the item scores and dividing by the number of items. The mean scores per item indicate the average response along the five-point Likert scale.

#### Physical barriers to performing family food tasks

To assess physical barriers to practising food-related tasks, participants were also asked if they had reliable access to adequate food preparation facilities (yes/no) and if they had any disabilities preventing them from planning, shopping and preparing meals (yes/no).

#### Interest in resources to support family food provision

Participants were asked to indicate their perception of the usefulness of a list of ten common support tools or resources (e.g. meal kits, shopping guides, cooking lessons, quick meal ideas and recipes). The response options included ‘already using’, ‘would like to use’, ‘maybe’ and ‘not interested’. A free text option was provided for participants to enter other tools or resources that would be helpful.

#### Sociodemographic details

Sociodemographic questions included indicators of socio-economic status (postcode, highest education level and employment status), marital status, date of birth, cultural background (country of birth, indigenous status and main language spoken at home), and number and ages of their children. Postcodes were categorised using the Index of Relative Socio-Economic Disadvantage and organised into quintiles (1 most disadvantaged to 5 least disadvantaged) to produce the Socio-Economic Indexes for Areas (SEIFA)^(28)^. SEIFA provides a measure of the socio-economic level of the geographic area. Fathers who did not live with the biological parent of their children were asked how often (days per month) they had caring responsibilities for their children.

Participants’ work conditions potentially impacting their availability in being involved in family food tasks were assessed. These included shift work requirements (yes/no) and frequency of travelling and staying away from home for work (none, less than once a month, 1–2 times per month, 3–4 times per month or more).

#### Survey validity and reliability

The survey was first piloted with a small convenience sample of people in the target group with a mix of demographic characteristics, including couple and single fathers, culturally diverse fathers, and a range of educational levels. Pilot participants provided feedback to clarify the wording and order of several questions. These changes were incorporated to improve the clarity and flow of the survey.

The tool measuring practices in family food tasks was previously validated among a predominantly female sample. We performed further confirmatory factor analysis to assess the fit of the original structure with the male sample in this study. The confirmatory factor analysis showed that the original factor structure did not perform well with our data (RMSEA 0·083; CFI 0·873; TLI 0·830; SRMR 0·059)^(29)^. It is likely that there are gender differences in conceptualisations regarding food literacy^(30)^, hence the poor fit among our male participants. Consequently, these items were not analysed as a scale but were described individually.

The CAFPAS tool had good construct and criterion validity, and internal reliability in the original study among a diverse sample of the US population^(27)^. Further confirmatory factor analysis showed that the tool had an acceptable fit for this study population (RMSEA 0·06; CFI 0·895; TLI 0·886; SRMR 0·064)^(29)^ and high internal consistencies (*α* 0·71–0·90)^(31)^.

The reliability of the survey across time was assessed through the test–retest method where a subset of sixty-six participants was invited to complete the survey around 2 weeks after first completion^(32)^. Cohen’s *κ* ^(33)^ was used to measure the degree of agreement between the two time points, and coefficients were interpreted using cut-off values suggested by Landis and Koch^(34)^.

The reliability of the measures of levels of responsibility in family food tasks and attitudes towards their responsibilities were mostly ‘substantial’ (*κ* 0·57–0·74); practices in family food tasks were mostly ‘moderate’ (*κ* 0·42–0·68); and perceived usefulness of tools to support food provision were ‘fair to moderate’ (0·32–0·51). The CAFPAS tool had ‘fair to moderate’ reliability (*κ* 0·25–0·58), except for one item showing ‘substantial’ reliability (*κ* 0·64).

#### Statistical analysis

Descriptive analyses were used to describe the proportion of perceived responsibilities and views of family food tasks, practices in family food tasks, mean scores on CAFPAS scale and subscales, and preferred tools to support family food tasks.

Ordered logistic regression was used to examine the relationships between perceived responsibilities in food tasks (‘food shopping’ and ‘meal preparation’) and key factors including sociodemographic characteristics (age, country of birth, education, employment status (full-time/other)), SEIFA, work conditions (shift work, frequency of working away from home (none/other)), number of children (1, 2, 3 or more), self-efficacy, attitude and structural barrier subscales. For ‘meal planning’, the assumption of proportional order could not be satisfied; hence, multinomial logistic regression was used with ‘little or no responsibility’ set as the referent group. Multiple linear regression was used to assess if there are any relationships between food agency and sociodemographic characteristics, work conditions, and the number of children.

All models were checked for collinearity (none detected). Statistical significance was set at 0·05. Data analyses were performed using Stata version 17.0 software.

## Results

### Participants’ characteristics

Four hundred and seventy-one fathers participated in the study, but thirty-six were excluded due to missing all demographic data (*n* 34) or providing duplicate responses (*n* 2). Complete responses were available for 435 participants, and their demographic characteristics are presented in Table 1. Participants were on average 36 years old (sd 5 years), tertiary-educated (58 %), working full time (78 %) and on average had one (45 %) or two children (47·2 %). Most participants were in partnered relationships (98 %), born in Australia (83 %) and spoke English at home (98 %). Over one in four (28 %) participants lived in two of the most disadvantaged SEIFA quintiles areas. Most participants did not do shift work (82 %) or travel regularly for work (76 %). All participants had reliable access to food preparation facilities, and none had physical barriers preventing them from being involved in food tasks.

**Table 1:** Participants’ characteristics (*n* 435)

Characteristics	Mean	sd
Paternal age, years	36	5
	*n*	%
Education level		
Below tertiary	184	42·3
Tertiary education or higher	251	57·7
Marital status		
Married	341	78·4
De facto relationship	87	20
Divorced/separated/widowed	7	1·6
Country of birth		
Australia	360	82·8
Other	75	17·2
Aboriginal and Torres Strait Islanders’ status		
Yes	8	1·8
No	427	98·2
Main spoken language at home		
English	424	97·5
Other	11	2·5
Socio-economic indexes for areas (SEIFA)		
1st quintile (most disadvantaged)	51	11·8
2nd quintile	69	15·9
3rd quintile	88	20·3
4th quintile	103	23·7
5th quintile (least disadvantaged)	123	28·3
Employment status		
Full time	337	77·5
Part time	63	14·5
Not working	35	8·05
Shift work (*n* 401)		
Yes	72	18
No	328	82
Regularly away from home (if employed)		
None	302	75·5
Less than once a month	57	14·3
1–2 times/month	29	7·3
3–4 times/month	12	3
Number of children		
1	194	44·7
2	205	47·2
3 or more	35	8·1
Children’s ages		
Expecting (child in utero)	94	13·2
0–6 months	80	11·3
7–12 months	83	11·7
13–24 months	123	17·3
2–3 years	201	28·3
4–5 years	130	18·3

### Perceptions and views of responsibilities in family food tasks

Between 75 and 77 % of participating fathers reported having equal or more responsibilities than their partner for family food tasks. Coronavirus pandemic restrictions did not impact the levels of responsibility on family food tasks for most (75 %) participants, while 21 % had increased and 5 % fewer responsibilities. Fathers’ level of responsibility varied with each task. More than 40 % of fathers reported having ‘all or most of the responsibilities’ for meal preparation (48 %), food shopping (46 %) and meal planning (41 %). Meal planning was the task with the highest proportion of fathers reporting shared (35 %) or little (25 %) responsibility.

Most participants were satisfied with their levels of responsibility for food shopping (81 %), meal planning (75 %) and meal preparation (72 %). Some fathers reported wanting more involvement in meal preparation (18 %), meal planning (17 %) and food shopping (13 %). A small proportion of fathers indicated they had too many responsibilities with meal preparation (10 %), meal planning (8 %) and food shopping (6 %). Detailed results are presented in Supplemental Tables 1(a) and (b).

### Practices in family food tasks

Table 2 details the self-reported frequency of family food tasks participants conducted in the last month. Tasks reported as practised most of the time or always were cooking healthy meals (87 %), cooking a variety of meals (81 %) and thinking about healthy choices when deciding what to eat (83 %). Many fathers also used a shopping list (74 %), planned meals (53 %) and planned meals to include most or all food groups (66 %). Tasks reported as practised never or sometimes were reading food labels including the nutrition information panel to make food choices (75–78 %), trying a new recipe (75 %) or changing recipes to make them healthier (71 %).

**Table 2:** Father self-reported frequency of performing family food tasks in the previous month

Items*	Never		Sometimes		Most of the time		Always	
	*n*	%	*n*	%	*n*	%	*n*	%
Plan family meals ahead of time	21	4·83	177	40·69	172	39·54	65	14·94
Make a list before you go shopping	28	6·44	83	19·08	140	32·18	184	42·30
Plan meals to include most/all food groups	33	7·59	117	26·90	202	46·44	83	19·08
Think about healthy choices when deciding what to eat	6	1·38	70	16·09	230	52·87	129	29·66
Manage the amount of money that can be spent on family food	63	14·48	127	29·20	145	33·33	100	22·99
Use the Nutrition Information Panel to make food choices	148	34·02	191	43·91	76	17·47	20	4·60
Use other parts of food labels to make food choices	76	17·47	251	57·70	87	20·00	21	4·83
Cook meals at home using healthy ingredients	3	0·69	53	12·18	298	68·51	81	18·62
Cook a variety of meals for the family	8	1·84	75	17·24	240	55·17	112	25·75
Try a new recipe	20	4·60	307	70·57	90	20·69	18	4·14
Change recipes to make them healthier	60	13·79	249	57·24	116	26·67	10	2·30

* Items from evaluation tool for food literacy programmes^(26)^.

### Food agency

The mean overall CAFPAS score and mean scores per item are presented in Table 3. Overall, the results indicated that participants had an above-average food agency (mean score per item 3·7 (range 1–5)) driven by a high self-efficacy in food provisioning and cooking abilities (mean score per item 4·1 (sd 0·5)), generally positive attitude towards food and cooking (mean score per item 3·4 (sd 0·6)), and some perception of structural barriers to food provisioning (mean score per item 3·13 (sd 0·7)). The Self-efficacy Subscale had the highest average score per item, followed by the Attitude Subscale and Structure Subscale.

**Table 3:** Mean scores of the cooking and food provisioning action scale (CAFPAS) and subscales^(27)^

	No. of items	Standardised mean scores	sd	Unstandardised mean scores per item*	sd
Full CAFPAS†	28	17·6	2·3	3·7	0·5
Self-efficacy subscale†	13	7·5	1	4·1	0·5
Attitude subscale†	10	5·6	1	3·4	0·6
Structure subscale‡	5	4·4	1	3·13	0·7

* The mean per item scores indicate the average response on the five-point Likert scale (1 – strongly disagree, 5 – strongly agree).

† Higher scores indicate higher food agency/self-efficacy or a more positive attitude.

‡ Higher score indicates a lower perception of structural barriers.

Descriptive results of the items in the Self-efficacy Subscale (see online Supplemental Table 2) indicated most participants (55–97 %) were confident in their ability to decide what to eat, shop for the ingredients and choose between similar ingredients, use the available kitchen equipment to prepare meals, prepare meals using purchased ingredients and troubleshoot cooking problems. The Attitude Subscale items showed that while 63–66 % of participants reported enjoying cooking for themselves and family and friends, there was ambivalence in choosing to cook if other people could prepare meals of equal standard. Similar ambivalence was shown in preference for time spent cooking over other activities. Time barriers associated with employment responsibilities (but less so with family or social responsibilities) were apparent. Half of the participants wished they had more time to plan meals, and 40 % of participants reported difficulties in finding enough time to produce the foods desired.

### The influence of food agency and sociodemographic factors on perceived responsibilities in food tasks

#### Meal planning

Fathers were more likely to have most or all meal planning responsibilities if they were older (RRR 1·08; 95 % CI (1·01, 1·15); *P* = 0·024), not working or working part-time (RRR = 5·60; 95 % CI (1·67, 18·75); *P* = 0·005), had higher self-efficacy scores (RRR = 1·20; 95 % CI (1·12, 1·29); *P* < 0·001), more positive attitudes (RRR 1·11; 95 % CI (1·03, 1·19); *P* = 0·007) and lower perception of structural barriers (1·10; 95 % CI (1·00, 1·21); *P* = 0·044). Fathers were less likely to have most of the meal planning responsibilities if they lived in moderately disadvantaged areas (SEIFA quintile 3) (RRR 0·38; 95 % CI (0·15, 0·97); *P* = 0·043) compared to those living in most disadvantaged areas (SEIFA quintiles 1–2). There were no statistically significant associations between having major responsibility for meal planning and country of birth, education level, the number of children, or whether fathers do shift work or travel away from home for work.

#### Food shopping

Fathers with higher self-efficacy scores had higher odds (OR 1·07; 95 % CI (1·03, 1·12); *P* < 0·001) of having most of the responsibilities in food shopping. Those living in moderately disadvantaged areas had lower odds (OR 0·56; 95 % CI (0·32, 0·996); *P* = 0·048) of having major responsibilities in food shopping compared to those living in most disadvantaged areas. The odds of having a major responsibility for food shopping were not influenced by age, country of birth, education level, employment status, number of children, shift work, travel away from home for work, level of perceived structural barriers and attitudes in food provisioning.

#### Meal preparation

Fathers had higher odds of having most responsibilities in preparing meals if they had higher self-efficacy scores (OR 1·14; 95 % CI (1·09, 1·20); *P* < 0·001), more positive attitude (OR 1·06; 95 % CI (1·01, 1·11); *P* = 0·028), and lower perception of structural barriers (OR 1·08; 95 % CI (1·01, 1·14); *P* = 0·021) and were working part-time or not working (OR 1·99; 95 % CI (1·09, 3·65); *P* = 0·025). The odds of having a major responsibility for meal preparation were not influenced by age, country of birth, education level, SEIFA, number of children, shift work or travel away from home for work. All regression models assessing responsibilities in each food task are detailed in Supplemental Table 3.

#### Sociodemographic factors influencing food agency

Higher food agency scores were significantly associated with working part-time or not working (*β* 4·51; 95 % CI (0·79, 8·23); *P* = 0·018) and older age (*β* 0·27; 95 % CI (0·00, 0·54); *P* = 0·047). Being born in countries other than Australia was associated with lower food agency (*β* −4·09; 95 % CI (−7·71, −0·48); *P* = 0·026). Further analyses on each subscale showed that older fathers had higher self-efficacy (*β* 0·15; 95 % CI (0·01, 0·30); *P* = 0·031) and fathers who worked part-time or not working had lower perceptions of structural barriers (*β* 1·22; 95 % CI (0·25, 2·21); *P* = 0·014). Fathers who were born outside Australia had lower self-efficacy (*β* −2·03, 95 % CI (−3·93, −0·14); *P* = 0·035) and poorer attitudes (*β* −1·89; 95 % CI (−3·48, −0·28); *P* = 0·021) compared to their Australian born counterparts. The overall regression model of the full food agency was statistically significant explaining about 3 % of the variation in food agency scores (adjusted *R* ^2^ 0·035). Detailed results of the regression models can be found in Supplemental Table 4.

#### Supports to facilitate fathers’ involvement in family food tasks

Most fathers reported they had not used the listed resources to support their involvement in family food tasks (see online Supplemental Table 5). The resources most fathers were using included online cooking lessons (14 %) and meal kits (11 %). The resources most fathers reported they would like to/may use included quick meal ideas and recipes (80 %), healthy eating guide (79 %), menu plan (67 %), shopping guide (62 %) and eating out food selection guide (61 %). Resources fathers expressed the least interest in using were shopping guides on pre-made meals (65 %), meal kits (57 %), face to face (56 %) or online (44 %) cooking lessons, and food budgeting tools (47 %).

Twenty participants provided further comments on resources. Resources that participants found helpful were recipes or cooking shows, and access to more convenient ways to shop such as online ordering and delivery services. Some participants commented wanting more guidance around providing and feeding children such as planning meals for toddlers, meal ideas and recipes for children, and ideas to cater for children with food allergies. Comments also showed that cost and lack of flexibility prohibited greater use of meal kits.

## Discussion

This is one of the first studies to examine how Australian fathers of young children contribute to healthy food provisioning at home, key promoters and barriers, and potential resources to facilitate fathers’ involvement in family food tasks. We found that most fathers shared at least half of the responsibilities to plan, shop, and cook meals, were confident, and satisfied with their food-related responsibilities. Many fathers also considered health when cooking and planning meals, but few fathers used nutrition and food labels when making food choices, tried new recipes or modified recipes to make them healthier. Study participants also had a relatively high food agency, which was associated with higher involvement in family food tasks. However, having perceived limited time due to work responsibilities negatively impacted food agency and involvement in food tasks, especially in meal planning and preparation. Participants indicated high interest in resources to support general and children-specific healthy meal planning, food shopping, and cooking such as quick meal ideas and recipes, shopping guide, and cooking lessons.

The level of perceived responsibilities in family food tasks among fathers in this study was almost double that previously reported in the literature. Studies in Australia and the USA have reported about 42–50 % of fathers with young children were highly involved in family food tasks^(35,36)^. Evidence suggests that fathers’ involvement in food tasks has increased over time, but mothers continue to bear the major responsibilities in food tasks^(37)^. The significantly higher proportion of fathers in our study who were highly involved in food tasks could be related to self-selection bias where fathers who are interested and engaged in health or food tasks were more likely to participate in the study. Also, data for this study were collected when various COVID-19 lockdowns were in place, which contributed to increased food-related responsibilities for some fathers. Compared to similarly aged (25–44 years) men in the 2021 Australian Census, our sample had higher proportions of fathers who were married (78 % *v*. 44 %), tertiary-educated (58 % *v*. 45 %), born in Australia (83 % *v*. 59 %) and spoke mainly English (98 % *v*. 70 %)^(38)^. There is some evidence suggesting that higher education is associated with more time spent on housework^(39)^. Despite the wide variation in levels of involvement, fathers with young children were engaged in healthy food provisioning which presents an early opportunity for nutrition and feeding interventions.

While fathers’ increased level of participation in food tasks is encouraging, their involvement appears to be secondary to mothers. It appears that while men are involved, women may be carrying a heavier cognitive load in managing overall household labour, including food-related tasks^(40)^. This is echoed in other studies which showed that Australian fathers regarded their contribution to family food as important and are committed to more equally sharing the tasks, but that their roles were limited to assisting due to a lack of time and cooking capabilities^(19)^. Our study also found lower participation in tasks that require a higher mental load or knowledge such as meal planning or food and nutrition label reading or recipe modification. The finding may suggest that fathers were comfortable contributing to managing family meals within a well-established food provisioning system, but it is unclear how well they could respond to deviations, for example, when their partner is unable to do food tasks or changes in children’s nutritional requirements or food preferences. In addition to supporting fathers’ involvement in food tasks, increasing literacy on food and nutrition may be important to support their resilience to environmental or social factors that threaten healthy food provisioning^(21)^.

Among fathers in this study, higher food agency and spending less time in paid employment were associated with higher perceived responsibilities in food tasks, particularly meal planning and preparation. Food agency constructs are likely related to individual capacity, opportunity and motivation which are common predictors of behaviour^(41)^. Another study reported similar findings showing positive associations between parental food agency and healthier cooking and eating practices^(42)^. Fathers in the current study had a relatively high food-related self-efficacy, which is in contrast to other studies among Australian fathers reporting a lack of confidence in preparing family meals^(19)^. This might be explained by a biased study sample and/or that there were other factors contributing to fathers’ food-related self-efficacy not explored in this study such as child fussy eating behaviour, the role of their partner in managing family food tasks and fathers’ experience in food tasks. Mastery and social modelling are key contributors to self-efficacy^(43)^. It is also likely that men who have engaged in food tasks prior to becoming fathers have had more time to experiment and develop their skills and mastery.

Time availability was an important overall theme in our findings related to both fathers’ food agency and involvement in family food tasks. Working part-time or being unemployed was a significant predictor of both food agency scores and fathers’ involvement in meal planning and meal preparation. Employment status may be a proxy for the time and flexibility available for fathers to contribute to these tasks. Greater involvement in these tasks over time may lead to higher self-efficacy and more positive attitudes, which in turn promotes continued involvement, creating a positive reinforcing cycle^(44)^. This is in line with literature examining the relationship between the length of paternity leave and fathers’ increased involvement in child-rearing tasks. Longitudinal studies have shown that longer periods of paternity leave are associated with more frequent responsibility and engagement of fathers in development and caretaking tasks in the first few years of their child’s life, and this is partially mediated by fathers’ attitudes^(45)^. This highlights the important influence of paternity leave and flexible work arrangements for fathers that allow greater shared care of child-rearing and other family tasks including healthy family food provisioning.

In addition to employment status, other sociodemographic factors influencing food agency scores were age and country of birth. Older fathers had high food agency scores, and this may reflect greater knowledge and skills with food-related tasks over time^(42)^. Lower food agency scores amongst fathers born outside of Australia may reflect a difference in gender roles associated with food provisioning tasks across cultures^(46)^. Immigrant men may need to prioritise employment given that they were often the principal applicant in the Australian skilled migration scheme^(47)^. As a result, women as secondary/spouse applicants by default may take on more responsibilities in the home^(47)^. This may be the case for immigrants from culturally and linguistically diverse backgrounds, as evidence showed these immigrant groups had the largest gender gap in housework^(48)^. Further research is needed to understand the household dynamics and healthy food provisioning within culturally and linguistically diverse families.

In this study of fathers who had a relatively high food agency, there was a high interest in using resources to support meal planning, shopping and cooking. Specifically, fathers were interested in resources to help with producing healthy food simply and quickly such as quick meal ideas and recipes and healthy eating guides. This finding is similar to a previous qualitative study of Australian fathers with young children reporting fathers’ desire to access evidence-based resources to support their children’s eating and physical activity^(20)^. Furthermore, given that few fathers in this study used food labels to guide food choices, adapted recipes to make them healthier or tried new recipes, targeted online resources^(49)^ on nutrition label reading and recipe adaptation may further enhance fathers’ capacities in healthy food provisioning^(50)^. Participants’ interests in using supporting resources present an opportunity for intervention that could be explored further. In addition, the finding that most fathers in this study were not interested in using commercial meal kits is of interest given the rapid increase in the meal kits market. In contrast to fathers’ views, research has indeed found that mothers report meal kits to be attractive as they reduced the cognitive load associated with planning and preparing healthy home-cooked meals^(51)^. Given the finding in our study that fathers tended not to engage in planning meals, it is perhaps not surprising that the value of meal kits was not embraced. Regardless, the potential for meal kits to provide an opportunity to support fathers’ participation in family meal provisioning provides an opportunity for further research.

### Strengths and limitations

A strength of this study was the use of more granular measures of family food practices enabling a more nuanced understanding of fathers’ practices related to meal planning, shopping and preparation. However, the items could not be reduced to factors representing concepts of meal planning/shopping/cooking and assess their relationships with key predictors. This reflects the lack of appropriate tools to measure practices on family food tasks among fathers, which represents an important area for future research.

The study sample under-represented fathers experiencing socio-economic disadvantage, Australian Indigenous people, and those with culturally and linguistically diverse backgrounds. Additionally, the self-selection recruitment method may have introduced a sample bias by attracting fathers who were interested and involved in food tasks. Evidence suggests that Facebook ^TM^ was the most feasible method to recruit ‘hard to reach’ populations and tended to result in a similarly representative sample as traditional recruitment methods^(52)^. To overcome selection bias, purposive sampling of underrepresented subgroups may be necessary, for example, through specific targeting strategies and/or tailoring the wording or image of the advertisement. Furthermore, all the measures in this study were self-reported and may be limited by social desirability and recall bias. Hence, the findings need to be interpreted and applied with care. Possible ways to validate self-reported measures of involvement in food tasks could be through: collecting and comparing data from couple dyads; conducting follow-up interviews with a subsample of fathers and their partners discussing the management of food tasks in the family; or through participant observations using wearable cameras. Finally, we acknowledge that in the contemporary society, there are diverse family types that may not always include a father. Further longitudinal studies with a more representative sample, including diverse family types, will be needed to confirm the direction and strength of the relationships between perceived responsibilities for family food tasks and food agency.

In summary, this study suggests that fathers with young children are receptive to making positive contributions to healthy home food provisioning. Increasing time availability and/or strategies to enable healthy food provisioning when time is limited appeared to be essential strategies in enhancing fathers’ food agency and involvement in healthy food provisioning at home.

## Conclusion

Fathers’ engagement and practices around food impact the family food environment with implications to young children’s dietary intake. This study found that fathers’ involvement in family food tasks was promoted by a greater sense of food agency, but a perceived lack of time availability due to employment prevented greater involvement and food agency. Given the cross-sectional design and likely non-representative sample, these findings need to be interpreted with care. Encouraging more flexible and balanced working arrangements and uptake of paternity leave as well as how food tasks can be better shared to promote greater food agency among fathers are likely to be important strategies.

## Supplementary material

For supplementary material accompanying this paper visit https://doi.org/10.1017/S1368980023001817
